# Learning to Stand: The Acceptability and Feasibility of Introducing Standing Desks into College Classrooms

**DOI:** 10.3390/ijerph13080823

**Published:** 2016-08-15

**Authors:** Roberto M. Benzo, Allene L. Gremaud, Matthew Jerome, Lucas J. Carr

**Affiliations:** Department of Health and Human Physiology, University of Iowa, Iowa City, IA 52240, USA; roberto-benzo@uiowa.edu (R.M.B.); allene-gremaud@uiowa.edu (A.L.G.); matt-jerome@uiowa.edu (M.J.)

**Keywords:** sedentary, physical activity, physical inactivity, standing desks, college classroom, standing desk

## Abstract

Prolonged sedentary behavior is an independent risk factor for multiple negative health outcomes. Evidence supports introducing standing desks into K-12 classrooms and work settings to reduce sitting time, but no studies have been conducted in the college classroom environment. The present study explored the acceptability and feasibility of introducing standing desks in college classrooms. A total of 993 students and 149 instructors completed a single online needs assessment survey. This cross-sectional study was conducted during the fall semester of 2015 at a large Midwestern University. The large majority of students (95%) reported they would prefer the option to stand in class. Most students (82.7%) reported they currently sit during their entire class time. Most students (76.6%) and instructors (86.6%) reported being in favor of introducing standing desks into college classrooms. More than half of students and instructors predicted having access to standing desks in class would improve student’s “physical health”, “attention”, and “restlessness”. Collectively, these findings support the acceptability of introducing standing desks in college classrooms. Future research is needed to test the feasibility, cost-effectiveness and efficacy of introducing standing desks in college classrooms. Such studies would be useful for informing institutional policies regarding classroom designs.

## 1. Introduction

Sedentary behaviors have become ubiquitous in today’s society, with many social and technological advances contributing to ever increasing sedentary behaviors [1,2]. A study conducted by Matthews et al. found the average individual (ages six and above) in the United States spends 7.7 h/day engaged in sedentary behaviors [3]. Sedentary behavior, defined as “any wakeful activity expending ≤1.5 METs in a reclining or sitting position”, has been associated with many adverse health effects [4,5]. Independent of physical activity, prolonged sedentary time has been associated with increased risk for several chronic diseases including cardiovascular disease, type II diabetes, and various cancers [5]. Previous studies have reported a negative association may also exist between sedentary behavior and brain health (decreased cognitive performance, mental distress, and dementia) [6,7]. Conversely, evidence suggests breaking up prolonged bouts of sedentary time may attenuate such negative health consequences [1]. A study conducted by Healy et al. found that increased interruptions of sedentary time was associated with better cardiometabolic risk factors (body mass index, waist circumference, triglycerides, and 2 h plasma glucose levels) [1]. With evidence suggesting potential health benefits from interrupting sedentary periods, it is now time to identify effective and sustainable interventions that reduce or interrupt sedentary behaviors amongst sedentary populations [1].

The transition to college has been identified as a critical window for decreased physical activity and increased sedentary behaviors [8]. The 2015 report from the National College Health Assessment estimates 55% of all college students fail to meet the recommended physical activity guidelines [9]. Evidence suggests college students spend as much as 30 h/week sedentary, with much of that time spent studying and sitting in class [10]. These numbers are particularly alarming considering physical activity levels continue to decline with age [11,12,13] and the steepest declines in physical activity have been found to occur between the ages of 12 and 18, the years immediately prior to college [14,15]. Additionally, physical activity habits of college students have been shown to track into adulthood [16]. For example, Telama et al. found reductions in physical activity, beginning at age 12, and tracking to 27 years of age [14]. A longitudinal study by Van Mechelen et al. found similar results in a population ranging from 13 to 27 years of age, with the steepest declines occurring throughout the teenage years [15]. These studies have been corroborated with more recent studies showing objectively-measured declines in physical activity from adolescence to young adulthood [17,18,19,20].

For the young adult who is already sedentary in the college years, progressive declines in physical activity over a lifetime increases the individual’s risk for the development of sedentary related chronic diseases later in life. With almost half of all young adults (18–24 years old) enrolled in college [21], there is a need to identify prevention-focused interventions that target the sedentary behaviors of this understudied population [22].

The Social-Ecological Model recognizes health behaviors including sedentary behaviors are influenced by factors at multiple levels of the environment including those found at the organizational level [23]. Standing desks have been demonstrated as an acceptable [24,25] and effective approach for reducing sitting time in both the K-12 classroom setting [26,27,28,29] and the occupational/work setting [30]. For example, a recent systematic review of eight standing desk intervention studies conducted in school-aged children found introducing standing desks increased student’s standing time (effect sizes: 0.38–0.71) and reduced sitting time (effect sizes: 0.27–0.49). A recent Cochrane review of 20 workplace interventions targeting workplace sitting found introducing standing desks reduced sitting between 30 and 120 min/day [30].

Based on the evidence supporting standing desks as an approach for reducing sitting time in K-12 classrooms and work settings, it is reasonable to assume introducing standing desks into college classrooms might also be effective for interrupting and/or reducing sedentary behaviors of college students. However, to date, no studies have explored the acceptability, feasibility or efficacy of introducing standing desks into the college classroom [2]. Therefore, the purpose of this study was to explore the acceptability and feasibility of introducing standing desks into traditional college classrooms. We hypothesized students and instructors would be in favor of introducing standing desks in college classrooms.

## 2. Methods

Both students and instructors at a single Midwestern university were recruited to complete a needs assessment survey. The survey explored beliefs and opinions of introducing standing desks in college classrooms. Participants of all races and ethnic backgrounds were recruited through a mass email, which included a link to an online survey. All data were collected during the month of September 2015. As an incentive to complete the survey, participants were entered into a drawing for one of ten $50 cash prizes. All participants completed an online informed consent prior to completing an online survey. All subjects gave their informed consent for participation before they commenced the study. The study was conducted in accordance with the Declaration of Helsinki, and the experimental protocol was approved by the University of Iowa Ethics Committee of Human Subjects Office Institutional Review Board (20150891).

Separate surveys were created for students and instructors. Both surveys inquired about general demographics (age, sex, race, ethnicity, academic department, full/part-time status). Students were also asked to self-report their weight, height (BMI was calculated using self-reported height and weight), smoking status, and sedentary behaviors and physical activity levels. Sedentary behaviors were estimated with the Rapid Assessment Disuse Index (RADI) which has been demonstrated as a valid and reliable measure of sedentary and light physical activity behaviors [31]. The RADI is made up of three questions which encompass two domains of daily activity (e.g., moving around and climbing stairs) on one of inactivity (e.g., sitting). Participants are asked to self-report their activity/inactivity for the last week, month and year resulting in a possible score of 3–15 for each item, and an overall score of 9 to 45. It has been recommended that individuals with a RADI score of 26 or higher (79% sensitivity, 63% specificity) should reduce sitting time and increase their lifestyle activity [31]. Higher RADI scores have been significantly correlated with increased sedentary time, fewer sedentary breaks, and decreased light physical activity [19]. Physical activity levels were estimated with a five-item single response physical activity survey (PA5) which has been demonstrated as a reliable and valid estimate of physical activity [32]. The PA5 has been shown to positively correlate with cardiorespiratory fitness (r = 0.57) in a previous study [32]. The PA5 dichotomizes participants into those who meet the physical activity guidelines (responses 4–5) for health benefits (i.e., accumulating 30 min or more of moderate intensity physical activity for a minimum of 5 days/week or vigorous intensity physical activity for a minimum of 3 days/week), and those who don’t meet the guidelines (responses 1–3) [32].

Both student and instructors were asked to report their opinions of introducing “standing desks” in college classrooms, but the surveys contained different questions. The term “standing desks” was used in the survey as opposed to other terms found in the literature which include sit-stand desks, height-adjustable desks, and stand-biased desks [2]. Students were asked the following questions: (1) the percentage of class time they currently spend sitting; (2) the percentage of class time they would stand if standing desks were available in their classrooms; (3) the degree to which a number of health and academic outcomes would change by having access to standing desks in class (1 = get worse; 2 = no change; 3 = get better); (4) whether they had ever taken a college class that had standing desks available (yes/no). Student favorability of introducing standing desks was determined based on responses to question 2 (percentage of class time students would spend standing if provided access to a standing desk). Students who reported they would stand more than 0% of class time if provided standing desks were identified as being “in favor of standing desks”, and those who reported they would sit 0% of class if provided standing desks were considered as “not in favor of standing desks”. This item was also used as a dependent variable in the linear regression model to determine predictors of student favorability.

Instructors were asked: (1) about their teaching experience at the college level (years); (2) whether they had ever taught a class that had standing desks (yes/no); (3) whether they would be in favor of introducing standing desks in their classrooms (yes/no); (4) the ideal location of standing desks (back row, middle row, front row, every row, end of row); (5) ideal classroom sizes for standing desks (small, medium, large, very large); and (6) the degree to which a number of health and academic outcomes would change for students if they had access to standing desks in class (1 = get worse; 2 = no change; 3 = get better). Instructor favorability (question 3) was used as the dependent variable in the logistical regression model aimed at identifying predictors of instructor favorability.

### Statistical Analysis

An estimated 31,387 students were enrolled at the university when the survey was administered. We estimated a sample size of 380 necessary to estimate the true population proportion with a required margin of error of 5% and a confidence interval of 95%. Out of 1997 students who opened the survey, a total 993 students (3.2% response rate) completed all items of the survey, reported to be an on-campus student, and were included in the final analysis. An estimated 5160 faculty and instructors were teaching courses at the university when the survey was administered. We estimated a sample size of 358 necessary to estimate the true population proportion with a required margin of error of 5% and a confidence interval of 95%. Instructors who reported teaching courses on campus and completed the survey in full were included for the data analysis. A total of 149 instructors met the inclusion criteria, resulting in a response rate of 2.9%. Descriptive statistics are presented in Table 1 using means, standard deviations, and percentages. Instructor and student summarized survey responses are reported in percentages (Table 2, Table 3 and Table 4). Ordinary least squares (OLS) regression analysis was used to examine student covariates predictive of increased favorability of standing desks (percentage of the time students would stand if standing desk were available). Coefficients in students’ OLS table (Table 5) represent percent time standing if standing desks were made available in college classrooms. The dependent variable for the student model was ordinal, but the variable was treated as continuous and ranged from 0% to 100%. Race was dichotomized into Whites or non-Whites in both student and instructor regression models. An average score for all perceived academic and health factors (ranging 1–3) was created using the following Equation:

Average Perceived Score = (# “get worse” responses * 1) + (# “no change responses” * 2) + (# “improvement responses” * 3)/total number responses

The perceived academic and health changes index measure was used to predict favorability of introducing standing desks for students and instructors in the OLS models (Table 5 and Table 6 respectively). A logistical regression model was used to examine whether instructors were in favor of introducing standing desks in college classrooms (Table 6). The dependent variable for the logistical model was a dichotomous categorical variable (1 = “in favor”, 0 = “not in favor”). Stata version 14.1 software was used to perform all statistical analyses.

## 3. Results

The student sample was on average 20.4 (4.1) years of age, 69.7% female, and had a self-reported body mass index (BMI) of 23.7 (4.4) kg/m^2^. Students were predominantly White (83.4%), more than half were freshmen status (51.5%), and the majority (94.8%) were full-time students. The instructor sample was on average 43.1 (13.7) years of age, 57.1% female, predominantly White, and 75.8% held full-time positions at the university. Instructors reported holding a variety of teaching positions from teaching assistants to full professors. Most students (62.8%) were categorized as living an inactive and sedentary lifestyle, resulting in an average RADI score of 27.5 (ranging from 3 to 45). Nearly half of students (46.8%) reported meeting the recommended physical activity guidelines [32].

Only 2.8% of students reported having used standing desks in college classrooms previously (Table 2). Most students (83%) reported they currently sit 100% of class time. More than half of all students (61%) reported a preference to alternate between sitting and standing during class compared to sitting or standing for the entire class period. If made available during class, 76% of students reported they would stand for at least 25% of class time.

Only 4% of instructors reported having taught courses previously in which standing desks were available (Table 3). Most instructors (85%) reported being in favor of introducing standing desks into their current classrooms. More than half of instructors reported standing desks would be well suited for medium (65.5%) and small (55.5%) sized classrooms while less than half felt they would be well suited for large (34.9%) or very large classes (19.5%). Instructors reported standing desks would best be located in either back rows (71%) or at the end of rows (41.6%).

Most students predicted either no change or positive changes (“get better”) in all academic and health factors (Table 4). Students reported physical health (56.2%), restlessness (53.0%), and attention (51.4%) as the factors most likely to “get better” while fatigue (33.7%), joint pain (31.0%), and restlessness (24.8%) were rated as the most likely factors to “get worse”. Similar to students, more than half of all instructors predicted either no change or positive changes (“get better”) for all academic and health factors presented (Table 4). Instructors reported physical health (67.1%), attention (66.4%), and engagement (61.7%) as the factors most likely to “get better” while fatigue (26.9%), restlessness (22.2%), and joint pain (13.4%) were rated as the factors most likely to “get worse” for students.

Neither race or sex predicted student favorability of standing desks (Table 5). Higher class status (*p* < 0.022), achieving physical activity guidelines (*p* < 0.002), past availability of standing desks in college classrooms (*p* < 0.018), and perceived improvements in academic and health factors (*p* < 0.000) was significantly associated with student favorability.

Neither age nor sex were significantly associated with instructor favorability (Table 6). However, race (White) and perceived positive academic and health changes for students predicted instructor favorability. Past use of standing desks in college classrooms was omitted from the model because all instructors (*n* = 6) who reported past use in their own classroom, were in favor of introducing them.

## 4. Discussion

This is the first study to explore the acceptability and feasibility of introducing standing desks in college classrooms among college students and instructors. The findings of this study suggest both students and instructors were largely in favor of introducing standing desks into college classrooms. This study builds upon previous studies which have explored the acceptability of standing desks in K-12 classrooms and occupational settings [25,33,34]. The high favorability of standing desks of students and instructors provides support for future interventions that test the efficacy of introducing standing desks in college classrooms on health and academic outcomes.

A small percentage of students (2.9%) and instructors (3%) reported previously taking or teaching college courses in which standing desks were available. While the present study was limited to a single academic institution, this finding suggests few college classrooms have included standing desks to date. This survey would need to be replicated at other universities to determine whether this is consistent with other institutions. The scarcity of standing desks in college classrooms likely contributed to most (83%) students reporting sitting for the entire duration of their college classes (i.e., without access to standing desks in class, students likely will not stand). Interestingly, only 23% of students reported they would still sit the entire class time if standing desks were made available. In other words, this data suggests the percentage of students who would sit the entire class period could decline by as much as 77% if standing desks were simply made available to all students. This suggests most students would prefer the opportunity to stand for at least a portion of their class time.

Previous studies conducted in K-12 classes have reported daily sitting time reductions of 59 to 64 min (9.4%–9.4%) per day following the introduction of standing desks [25,34,35]. It should be noted the typical college student does not spend the entire day in the same classroom, but rather takes classes in a variety of different rooms over the course of the day. Further, most college classes range from 50 min to 3 h in duration. More than half of all students (61%) in this study reported they would stand between 25% and 50% of class time if provided a standing desk. For the traditional 50-min class taught three times per week, a 25% reduction in sitting would equate to 12.5 min less sitting per class. Based on this conservative estimate, full institutional adoption of standing desks in all college classrooms could have a significant and wide reaching impact on the sedentary behaviors of college students. Future interventions are needed to confirm these projections.

Over half of students reported student’s physical health, attention in class, and restlessness would “get better” if standing desks were made available in college classrooms. Less than one third of students reported any of the heath or academic outcomes would “get worse” by introducing standing desks. Collectively, these finding suggest students feel the benefits of introducing standing desks outweigh the potential risks.

Studies exploring the effect of standing and use of standing desks on academic and health benefits are mixed [2]. A study by Mehta et al. found long-term use of standing desks was associated with significant improvements in cognitive function (i.e., executive function and working memory capabilities) amongst high school students. However, a recent review of 11 studies on the effects of standing desks on learning outcomes concluded that standing desks do not improve nor impair learning of elementary students [36]. Future interventions amongst college students are needed to explore the efficacy of introducing standing desks on academic outcomes. This information is critical for informing future policy decisions regarding the introduction of standing desks in college classrooms.

More than half of all instructors predicted student’s physical health, attention, restlessness, engagement, and boredom would improve if they had access to standing desks. More positive expectations for these outcomes was predictive of instructor favorability. Understanding the instructor’s favorability of standing desks is important prior to introducing these desks into college classrooms as they represent a key stakeholder in this decision. For example, if instructors felt introducing standing desks would interrupt the classroom dynamic, this would present a major barrier to introducing standing desks. In terms of the feasibility of introducing standing desks to college classrooms, instructors reported standing desks would be most suitable for small and medium sized classrooms and less suitable in large classrooms. Instructors also reported standing desks would be best located in the back of the room or end of rows. This information should be taken into account to help guide the placement of standing desks in college classrooms to minimize disruption and negative consequences.

The three most common factors perceived to get worse, by both instructors and students include fatigue, restlessness and joint pain. These findings suggest that standing desks should be accompanied by stools that allow students to transition between sitting and standing when they get tired or feel any musculoskeletal discomfort. Future interventions should include an orientation that describes the proper use of standing desks in class.

The two most influential predictors of student favorability were “past exposure to standing desks in college classrooms” and “perceived improvements in academic and health outcomes“. Given past exposure to standing desks predicted favorability, it is possible exposing college students to standing desks in class could prepare them to use or even expect standing desks when they enter the workforce later in life. The finding on perceived improvements in academic and health outcomes supports the inclusion of an educational component to future interventions which informs students of the potential academic and/or health benefits of standing compared to sitting.

This study had several limitations that should be considered. First the study was conducted at a single university and findings may not be generalizable to all universities. For instance, given the primarily White population included in this study, there is a need to replicate this survey amongst a more diverse population. More research is needed to further explore the feasibility of introducing standing desks in college classrooms as the present study did not fully address this topic. Future studies will need to explore the cost-effectiveness of this approach and explore the opinions of administrators in charge making furniture purchasing decisions at the college level. All data was self-reported and may be subject to recall bias. Finally, we did not achieve the necessary number of instructor responses to power the analysis. Despite these limitations, this was the first study to explore the acceptability and feasibility of introducing standing desks in college classrooms. Other strengths include a large sample that included both students and instructors.

## 5. Conclusions

The findings of this study indicate most students (83%) currently sit for the entirety of their college classes due in large part to the lack of access to standing desk options. The large majority of students (95%) reported they would prefer the option to stand in class. More than 75% of students and instructors reported being in favor of introducing standing desks into college classrooms. More than half of student and instructors predicted having access to standing desks in class would improve student’s physical health, attention in class, and restlessness in class. Interventions are needed to test the efficacy of introducing standing desks in college classrooms on sitting/standing behaviors as well as health and academic outcomes of college students. Feasibility studies are also needed to fully explore the cost-effectiveness of introducing standing desks. These efforts are critical for informing institutional policies regarding classroom designs for public health.

## Figures and Tables

**Table 1:** Baseline characteristics for students and instructors expressed in means (standard deviation) or percentages.

Descriptive	Students (*n* = 993)	Instructors (*n* = 149)
Age (years)	20.4 (4.1)	43.1 (13.7)
Female	69.7%	57.1%
Average BMI	23.7 (4.4)	N/A
Underweight	5.34%	N/A
Normalweight	66.77%	N/A
Overweight	19.44%	N/A
Obese	8.46%	N/A
Ethnicity		
Hispanic/Latino	7.2%	2.0%
Not Hispanic/Latino	90.7%	95.3%
Don’t Know/Refuse	2.1%	2.7%
Race		
American Indian/Alaskan Native	0.3%	0%
Asian	8.8%	4.7%
Native Hawaiian/Pacific Islander	0.4%	0%
Black	2.2%	0.7%
White	83.4%	88.6%
Other	2.4%	3.4%
Don’t Know/Refuse	2.5%	2.7%
Student Class Status		
Freshmen	51.5%	
Sophomore	11.1%	
Junior	9.2%	
Senior	10.3%	
Graduate Student	18.0%	
Full-time Student (%)	94.8%	
Instructor Title		
Graduate Teaching Assistant		12.1%
Lecturer		24.8%
Assistant Professor		20.8%
Associate Professor		18.8%
Professor		17.5%
Full-time Employee (%)		75.8%
RADI		
Active lifestyle	37.2%	N/A
Inactive lifestyle	62.8%	N/A
Average Score	27.5 (5.6)	N/A
Physical Activity Level (PA5) ^1^		
Met physical activity guidelines	46.8%	N/A
Did not meet physical activity guidelines	54.2%	N/A
Average Score	3.6 (0.9)	N/A

N/A: Not Available; ^1^ Five-item single response physical activity survey (PA5); Data was reported percentages, means, and standard deviations in parenthesis.

**Table 2:** Student’s perceived acceptability of standing desks.

Questions	Students (*n* = 993)
Have you ever taken a college class in which standing desks options were made available? (% Yes)	2.8%
On average, what percent of class time do you currently spend standing?	
0% of the time	82.9%
25% of the time	11.4%
50% of the time	1.9%
75% of the time	1.2%
100% of the time	2.6%
If given the option by your instructor would you prefer to sit or stand during class?	
Sit entire class time	34.5%
Sit part of the time and stand part of the time	60.8%
Stand entire class time	4.6%
* If standing desks were made available in a class you were taking, what percentage of class time do you predict you would stand on average?	
0% of the time	23.4%
25% of the time	34.5%
50% of the time	26.2%
75% of the time	12.3%
100% of the time	3.6%

Student data was reported as percentages.* Students’ index measure for being in favor of introducing standing desks in college classroom.

**Table 3:** Instructor’s perceived acceptability of introducing standing desks.

Instructor Survey Questions	Instructors (*n* = 149)
Have you ever taught college classes where standing desks where available? (%Yes)	4.0%
Would you be in favor of making standing desks available in classes you teach? (%Yes)	86.6%
How many years have you been teaching courses at the current university?	
1–5 years	56.4%
6–10 years	13.4%
11–15 years	8.1%
16+ years	22.2%
What class room size do you think standing desks would be well suited for?	
Small Class	61.7%
Medium Class (16–40)	67.1%
Large Class (41–100)	34.9%
Very large class (100+ students)	19.5%
Where would you prefer standing desks be located?	
Back rows	71.0%
Middle rows	6.0%
Front rows	0.0%
End of rows	41.6%
Every row	10.1%

Instructor data was reported as percentages.

**Table 4:** Students’ and instructors’ predicted changes in health and academic outcomes if standing desks were made available in college classrooms.

Factor (Value)	Students (*n* = 993)			Instructors (*n* = 149)		
	Get Worse (1)	No Change (2)	Get Better (3)	Get Worse (1)	No Change (2)	Get Better (3)
Physical Health	2.3	41.7	56.0	2.0	30.9	67.1
Joint Pain	31.0	46.8	22.4	13.4	45.0	41.6
Fatigue	33.8	28.7	37.5	26.9	35.6	37.6
Attention	18.2	30.8	51.0	6.0	27.5	66.4
Restlessness	24.8	22.1	53.1	22.2	24.2	53.7
Academic Performance	8.1	54.8	37.2	4.0	58.4	37.6
Engagement	8.8	42.6	48.6	5.4	32.9	61.7
Boredom	7.6	48.1	44.3	5.4	44.3	50.3

All results are presented as percentages. Improvement refers to the average perceived improvement across all measures.

**Table 5:** Model 1: Linear regression model to predict student favorability of introducing standing desks in college classroom.

Predictor Variable	Coefficients (Standard Error)	*p*-Value
Female ^1^	−0.083 (1.557)	0.958
White ^2^	1.315 (1.948)	0.500
Student Class Status	1.082 (0.471)	0.022 *
Meet PA Guidelines (PA5) ^3^	4.473 (1.463)	0.002 *
Inactive Lifestyle (RADI) ^4^	−2.291 (1.563)	0.143
Sit Stand Desk History ^5^	10.142 (4.289)	0.018 *
Perceived Academic and Health Improvement ^6^	30.967 (1.507)	0.000 **
Constant	−40.165 (4.199)	0.000 **
*Number of observations*	993	
*F-value*	67.84	
*Prob > F*	0.000	
*R-Squared*	0.3253	
*Adjusted R-Squared*	0.3205	
*Root MSE*	22.271	

^1^ Compared with males; ^2^ Compared to all other races; ^3^ Compared to those who do not meet the physical activity guidelines set by the CDC and ACSM; ^4^ Compared with individuals who score less than a 26 (active); ^5^ Compared with those that have had only been sit-desks available in college classrooms; ^6^ Index measure is the average score for all observations of academic and health factors presented in Table 4 (e.g., Physical health, focus, joint pain, etc.); * *p* < 0.05; ** *p* < 0.01; (two-tailed test).

**Table 6:** Model 2: Logistic regression model to predict instructor favorability of introducing standing desks in college classrooms.

Predictor Variable	Odds Ratio (Standard Error)	*p*-Value
Age	0.961 (0.025)	0.121
Female ^1^	1.22 (0.833)	0.769
White ^2^	5.076 (4.163)	0.048 *
Perceived Academic and Health Improvement ^3^	322.320 (415.185)	0.000 **
Constant	0.000 (0.000)	0.000 **
*Number of observations*	149	
*Logistic Regression Chi Squared*	56.93	
*Prob > Chi Squared*	0.000	
*Pseudo R-Squared*	0.484	

^1^ Compared with males; ^2^ Compared to all other races; ^3^ Change Mean: Index measure is the average score for all observations of academic and health factors presented in Table 4 (e.g., Physical health, focus, joint pain, etc.); * *p* < 0.05; ** *p* < 0.01; (two-tailed test).
